# MRI-Derived Tumor Characteristics As Prognostic Indicators in Prostate Cancer With Bone Metastases: A Preliminary Study

**DOI:** 10.7759/cureus.86829

**Published:** 2025-06-26

**Authors:** Hiroaki Koyama, Ryo Kurokawa, Takuya Fujimoto, Osamu Abe

**Affiliations:** 1 Radiology, Graduate School of Medicine, The University of Tokyo, Tokyo, JPN

**Keywords:** apparent diffusion coefficient, bone metastasis, magnetic resonance imaging, prognosis, prostate cancer

## Abstract

Background

While magnetic resonance imaging (MRI) is well-established for local assessment of prostate cancer, its prognostic utility in the metastatic setting remains poorly defined. We investigated whether pretreatment MRI findings can predict survival and disease progression in prostate cancer patients with bone metastases.

Methods

Of the 2,314 patients with pathologically proven prostate cancer between 2014 and 2021 in our hospital, this retrospective study finally included 19 patients with bone metastases who underwent prostate MRI within six months before diagnosis. Clinical and radiological parameters were analyzed, including Gleason score, prostate-specific antigen (PSA) density, clinical T stage, maximum tumor diameter (MTD), and normalized mean apparent diffusion coefficient (nADCmean). Time-dependent receiver operating characteristic curve analysis was used to determine the optimal cut-points for continuous variables with p-values <0.2. Univariate survival and disease progression analyses were performed using the Kaplan-Meier method and the log-rank test. Univariate Cox proportional hazards regression analyses were performed to estimate the hazard ratio.

Results

The median age was 69 years (range, 54-91 years). Gleason scores were 4 +4 = 8, 4 + 5 = 9, 5 + 4 = 9, and 5 + 5 = 10 in 12, three, three, and one patients, respectively. Median follow-up duration was 45 months. Seven patients died, and 12 experienced disease progression. MTD ≥49 mm was significantly associated with shorter overall survival compared to MTD <49 mm (median survival, 51 vs 95 months; p = 0.015). A higher nADCmean (≥0.19) was a significant prognostic factor for disease progression (p = 0.031). Gleason score, PSA density, and clinical T stage did not significantly discriminate outcomes in this metastatic cohort.

Conclusions

In prostate cancer patients with bone metastases, larger maximum tumor diameter on pretreatment MRI predicts poorer overall survival and may be more useful than clinical T stage for prognostic stratification. Higher normalized apparent diffusion coefficient (ADC) was associated with disease progression. Histopathological characteristics that raise ADC despite high Gleason score such as relatively low cell density, heterogeneous morphology, and cribriform pattern might have led to this result. These MRI-derived parameters could help guide treatment planning and risk assessment in metastatic prostate cancer. However, further studies are necessary to validate these findings due to the limitation of small sample size in this study.

## Introduction

Prostate cancer (PCa) represents the most commonly diagnosed malignancy among men across 112 out of 185 countries globally, with nearly 1.4 million newly diagnosed cases and 375,000 fatalities recorded worldwide in 2020 [1]. While PCa patients lacking bone metastasis (BM) demonstrate three- and five-year survival rates of 98.43% and 97.28%, respectively [2], approximately 3% of patients present with BM at initial diagnosis [3], and these patients experience substantially reduced survival rates declining to 47.70% and 32.42% [2].

Magnetic resonance imaging (MRI) of the prostate has proven valuable for identifying clinically significant PCa [4-7]. The Prostate Imaging Reporting and Data System (PI-RADS) [8] has gained widespread adoption in clinical settings as an instrument for risk stratification and standardized reporting enhancement [5], with additional reports indicating its ability to predict postoperative outcomes [9]. Clinical T staging correlates with biochemical recurrence, systemic disease progression, and cancer-related mortality following radical prostatectomy [10,11]. MRI demonstrates moderate to high diagnostic accuracy in predicting extraprostatic extension corresponding to T3a stage disease [12] and forecasts seminal vesicle infiltration equivalent to T3b stage disease [13]. Consequently, MRI can impact initial therapeutic planning and risk assessment.

Although MRI is well-established for local disease evaluation, its prognostic value in metastatic contexts remains less well-defined. Theoretically, aggressive primary tumor characteristics on MRI could indicate poorer outcomes even in systemic disease scenarios. For example, apparent diffusion coefficient (ADC) demonstrates negative correlation with Gleason score (GS) and cellular density [14-18]. Associations between ADC values and D'Amico clinical risk classifications as well as Ki-67 positivity rates in PCa have been documented [17,18]. Maximum tumor diameter (MTD) measured on MRI correlates with extraprostatic extension, positive surgical margins, seminal vesicle invasion, lymph node metastasis, and biochemical recurrence following radical prostatectomy [19-21].

Nevertheless, whether these MRI parameters can predict survival outcomes in patients presenting with metastatic disease at diagnosis remains poorly established. Considering this knowledge deficit, we conducted a retrospective investigation to examine the prognostic value of pretreatment MRI findings in PCa patients presenting with BMs.

## Materials and methods

The study was approved by the Research Ethics Committee of the Faculty of Medicine of the University of Tokyo, Tokyo, Japan (approval number: 2561-(28)). The need for informed consent was waived by the Research Ethics Committee of the Faculty of Medicine of the University of Tokyo due to the retrospective study design. Data were acquired in compliance with all applicable regulations of the Health Insurance Portability and Accountability Act, and all methods were performed in accordance with relevant guidelines and regulations. Data were de-identified prior to any analysis.

Patients

We performed a patient search using our hospital’s database. Between 2014 and 2021, 2,314 patients with pathologically proven PCa were identified in our hospital database. The following inclusion and exclusion criteria were used for patient selection. Inclusion criteria were as follows: prostate MRI was performed within six months before pathological diagnosis, body computed tomography (CT) was performed within six months before or after pathological diagnosis of PCa, and BMs were detected on CT examination, treated with hormone therapy including surgical castration, bicalutamide, flutamide, enzalutamide, abiraterone acetate, estramustine phosphate sodium hydrate, degarelix acetate, leuprorelin acetate, goserelin acetate, and ethinylestradiol, chemotherapy including docetaxel hydrate and cabazitaxel acetonate, or radiation therapy including radium-223 dichloride, and followed up at our hospital after diagnosis. Exclusion criteria were as follows: patients with concomitant or history of malignancy other than PCa, treatment was started prior to prostate MRI, BMs were detected on CT after the beginning of treatment, and treated with radical prostatectomy or radical radiotherapy.

Imaging acquisition

MRI was performed using 1.5T or 3T scanners. T2-weighted images were acquired using the following parameters: turbo spin echo; repetition time, 2,000-9,952.7 ms; echo time, 79-203 ms; flip angle, 90-160°; and resolution, 0.312 × 0.312-0.938 × 0.938 mm^2^. Diffusion-weighted images were acquired using the following parameters: repetition time, 3,209.3-16,300 ms; echo time, 66.6-95 ms; resolution, 0.885 × 0.885-3.017 × 3.017 mm^2^; and b values, at least (0 or 50 s/mm^2^) and (800, 1,000, or 1,500 s/mm^2^). The ADC maps were automatically generated by the implemented software.

Definition of terms

Prostate-specific antigen (PSA) density was defined as PSA divided by prostate volume, which was calculated as width × height × length × 0.52 [22]. Normalized mean apparent diffusion coefficient (nADCmean) was calculated by dividing the mean ADC of PCa by the mean ADC of the internal obturator muscle [23]. Disease progression was defined as biochemical or radiographic progression. Biochemical progression was defined as a 25% increase above the nadir and a minimum absolute level of 2 ng/mL, confirmed by two consecutive measurements at least three weeks apart [24].

Imaging analysis

All MRI and CT examinations were analyzed by two board-certified radiologists by consensus. They were blinded to the patients’ clinical and pathological information, except that they were diagnosed with PCa. The prostate size for calculating PSA density, clinical T stage, MTD, and nADCmean of PCa was evaluated using MRI. MTD was measured on axial, coronal, or sagittal T2-weighted images. ADC was measured by placing a circular region-of-interest, as large as possible, on the targeted areas while carefully avoiding the rim and vessels. Osteoblastic, osteolytic, or mixed bone lesions on CT images excluding mimickers of BMs such as degeneration, trauma, infection, and bone island were diagnosed as BMs.

Statistical analysis

Univariate survival and disease progression analyses were performed using the log-rank test and Kaplan-Meier methods. Continuous variables were divided by the median values. Time-dependent receiver operating characteristic (ROC) curve analysis was used to optimize the cut-point for continuous variables with p-values < 0.2. Univariate Cox proportional hazards regression analysis for each variable was performed to estimate the hazard ratio (HR). Statistical significance was set at p < 0.05. All statistical analyses were performed using the R software (version 4.3.1 or 4.5.0; R Foundation for Statistical Computing, Vienna, Austria).

## Results

**Figure 1:**
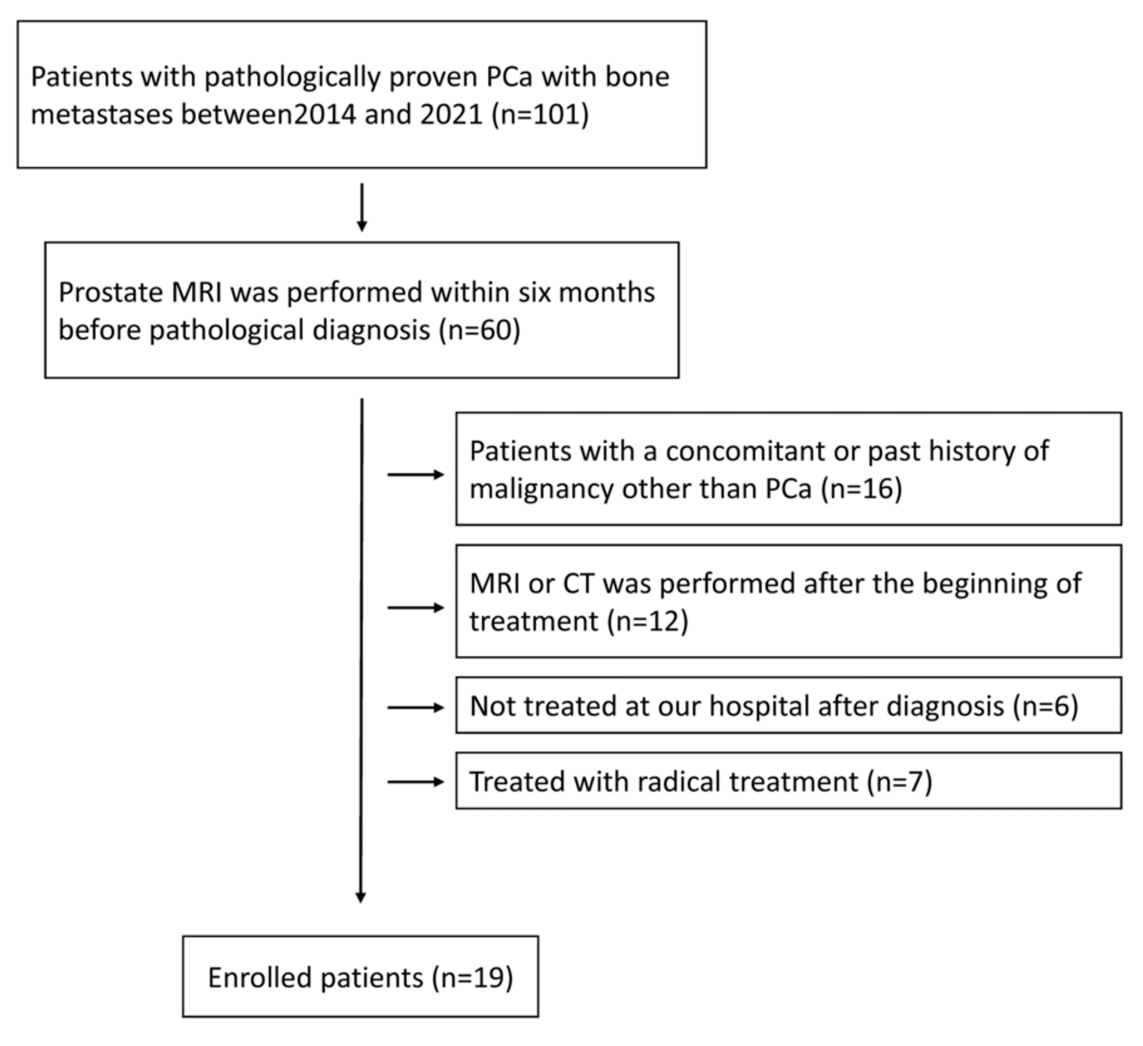
Flowchart of patient selection PCa: prostate cancer.

Of the 2,314 patients diagnosed with PCa between 2014 and 2021, 101 had BMs, and 60 patients received prostate MRI within six months before pathological diagnosis. After excluding patients with a history of malignancy other than PCa (n = 16), those for whom treatment had already started at the time of MRI or CT (n = 12), patients who were not treated at our hospital after diagnosis (n = 6), and treated with radical treatment (n = 7), 19 patients were included in the present study (Figure 1).

Patient characteristics

Patients' characteristics are presented in Table 1. The median age at diagnosis of the patient cohort was 69 years (range, 54-91 years). GS was 4 + 4 = 8 in 12 patients, 4 + 5 = 9 in three patients, 5 + 4 = 9 in three patients, and 5 + 5 = 10 in one patient. Median PSA density was 1.8 ng/mL/cm^3^ (range, 0.15-401 ng/mL/cm^3^). Clinical T stage was 3a in three patients, 3b in six patients, and 4 in 10 patients. Median MTD was 42 mm (range, 26-99 mm). Median nADCmean was 0.26 (range, 0.15-0.47). Median follow-up duration was 45 months (range, 18-95 months), and seven patients died after a median follow-up of 33 months (range, 18-95 months). Twelve patients experienced disease progression after a median follow-up of 14.5 months (range, 7-72 months).

**Table 1:** Patients' characteristics IQR, interquartile range; PSA, prostate-specific antigen; nADCmean, mean normalized apparent diffusion coefficient.

Patients' characteristics	Value
Age at diagnosis (median years, range, IQR)	69 (54-91, 66-80)
Gleason score (number of patients)	
8 (4 + 4)	12
9	6
(4 + 5)	3
(5 + 4)	3
10 (5 + 5)	1
PSA density (median ng/mL/cm^3^, range, IQR)	1.8 (0.15-401, 0.76-9.2)
Clinical T stage (number of patients)	
3a	3
3b	6
4	10
Maximum tumor diameter (median mm, range, IQR)	42 (26-99, 34-51)
nADCmean (median, range, IQR)	0.26 (0.15-0.47, 0.19-0.30)

Clinical and radiological analyses

**Figure 2:**
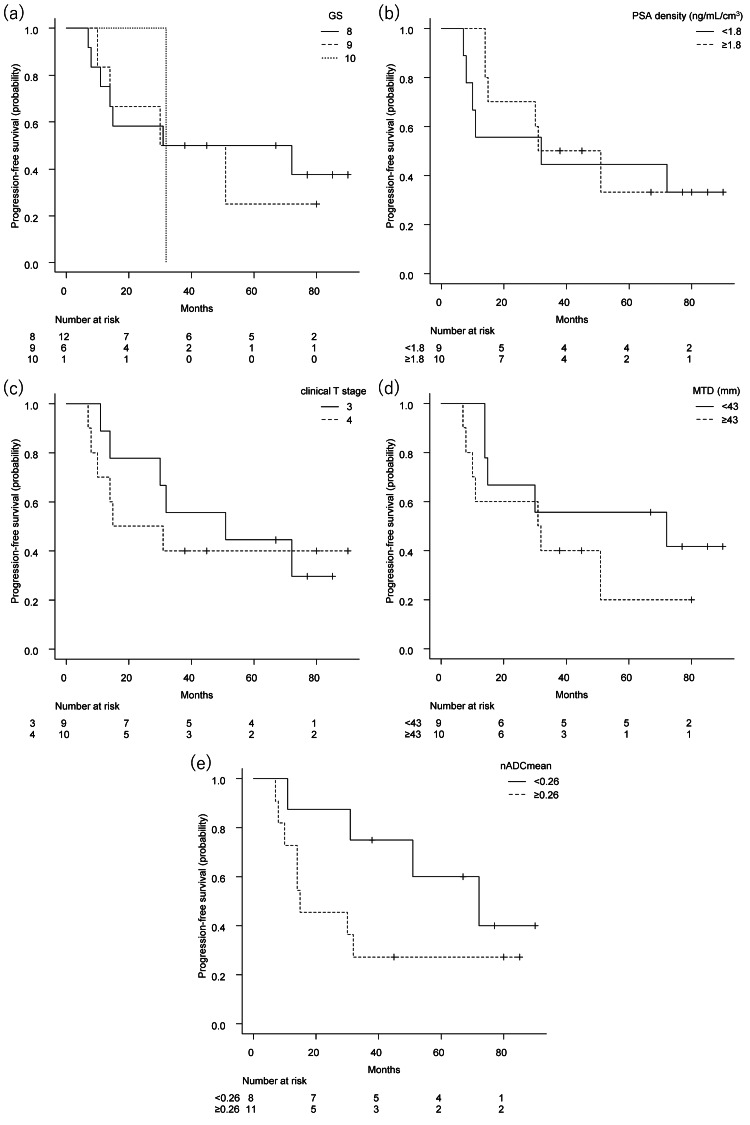
Kaplan-Meier curve of progression-free survival Kaplan-Meier curve of progression-free survival by Gleason score (a, p = 0.91), prostate-specific antigen density (b, p = 0.76), clinical T stage (c, p = 0.67), maximum tumor diameter (d, p = 0.29), and normalized mean apparent diffusion coefficient (e, p = 0.15). GS, Gleason score; PSA density, prostate-specific antigen density; MTD, maximum tumor diameter; nADCmean, normalized mean apparent diffusion coefficient.

**Figure 3:**
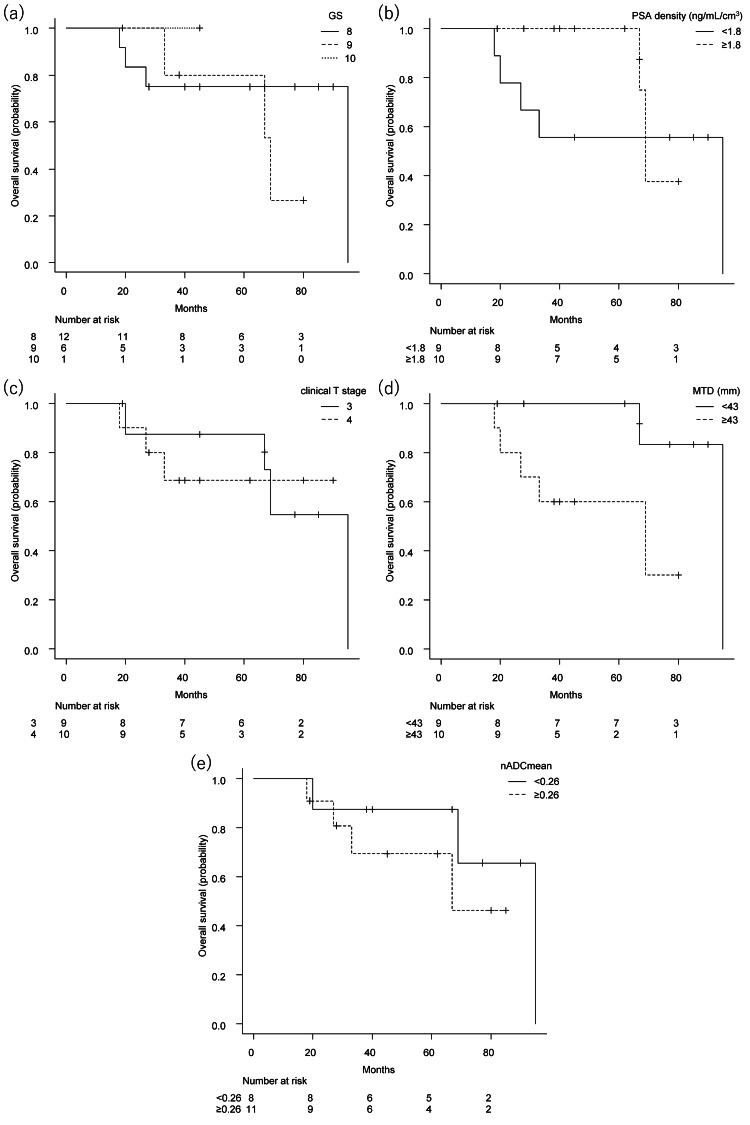
Kaplan-Meier curve of overall survival Kaplan-Meier curve of overall survival by Gleason score (a, p = 0.63), prostate-specific antigen density (b, p = 0.40), clinical T stage (c, p = 0.80), maximum tumor diameter (d, p = 0.047*), and normalized mean apparent diffusion coefficient (e, p = 0.35). *Statistically significant. GS, Gleason score; PSA density, prostate-specific antigen density; MTD, maximum tumor diameter; nADCmean, normalized mean apparent diffusion coefficient.

Figures 2, 3 show Kaplan-Meier curves of progression-free survival and overall survival, respectively. GS, PSA density, or clinical T stage was not a discriminator of outcome in this cohort.

**Figure 4:**
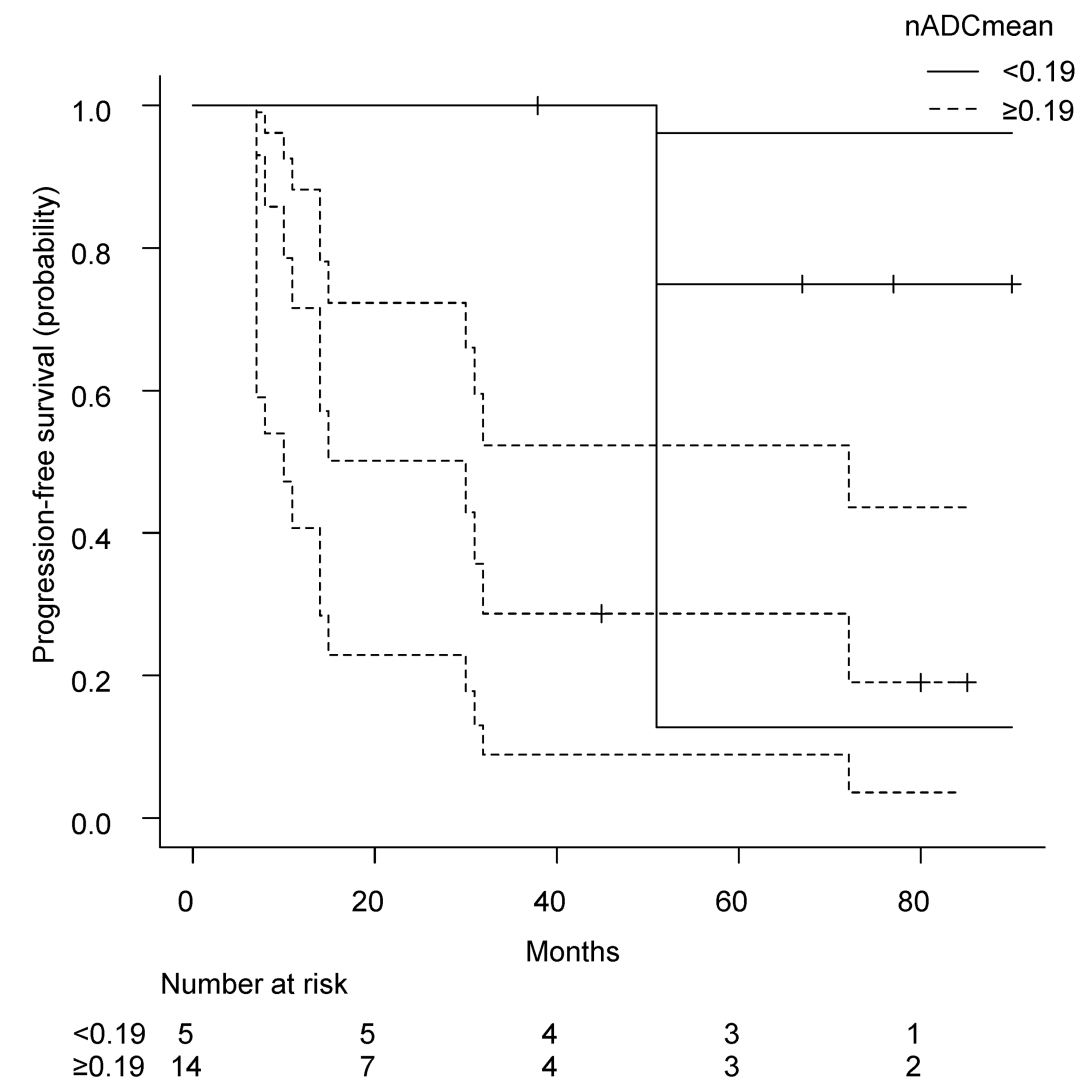
Kaplan-Meier curve of progression-free survival Kaplan-Meier curve with 95% confidence interval of progression-free survival by optimal cut-point of normalized mean apparent diffusion coefficient (p = 0.031*). *Statistically significant. nADCmean, normalized mean apparent diffusion coefficient.

**Figure 5:**
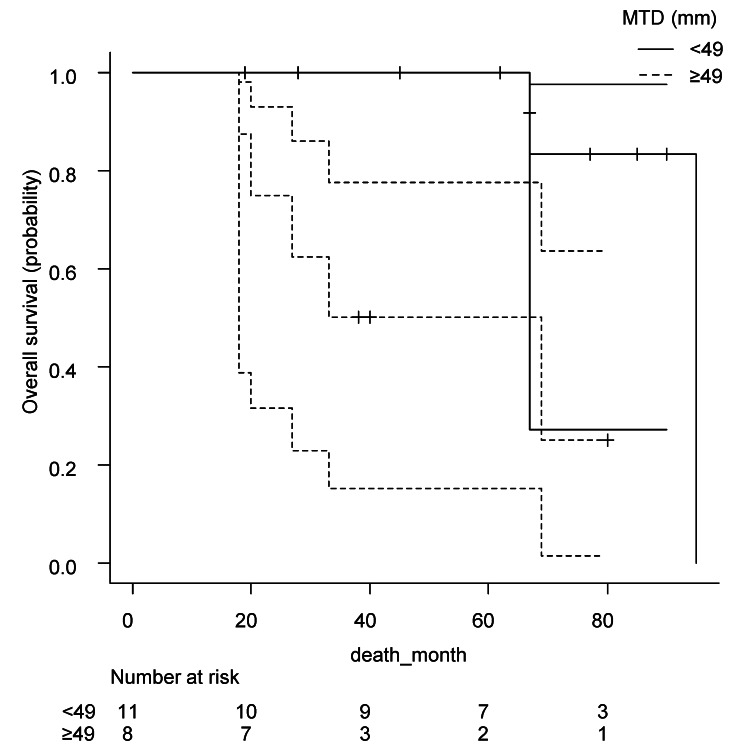
Kaplan-Meier curve of overall survival Kaplan-Meier curve with 95% confidence interval of overall survival by optimal cut-point of maximum tumor diameter (p = 0.015*). *Statistically significant. MTD, maximum tumor diameter.

Time-dependent ROC curve analyses with regard to three-year survival status and disease progression were completed to identify the optimal cut-points of MTD for survival and nADCmean for disease progression. The ideal cut-points were as follows: MTD ≥49mm for survival, and nADCmean ≥0.19 for disease progression. Area under the curve and 95% confidence interval (95% CI) were 0.912 (0.7781-1.0467) and 0.767 (0.5419-0.9914), respectively. We performed univariate analyses with these cut-points (Figures 4, 5).

nADCmean showed minimal changes, such that a 1-unit change resulted in a disproportionately large HR. Therefore, nADCmean was multiplied by 100 before inclusion in the Cox proportional hazards regression analysis. The results of univariate Cox proportional hazards regression analyses were shown in Tables 2, 3.

**Table 2:** Univariate Cox proportional hazards regression analyses for progression-free survival *Statistically significant. HR, hazard ratio; CI, confidence interval; PSA, prostate-specific antigen; nADCmean, mean normalized apparent diffusion coefficient.

	HR (95% CI)	p-value
Gleason score	1.217 (0.497-2.982)	0.6669
PSA density	1.002 (0.9966-1.008)	0.4477
Clinical T stage	1.275 (0.4076-3.985)	0.6767
Maximum tumor diameter	1.045 (1.007-1.084)	0.01856*
nADCmean	1.068 (0.994-1.147)	0.07249

**Table 3:** Univariate Cox proportional hazards regression analyses for overall survival *Statistically significant. HR, hazard ratio; CI, confidence interval; PSA, prostate-specific antigen; nADCmean, mean normalized apparent diffusion coefficient.

	HR (95% CI)	p-value
Gleason score	1.288 (0.3424-4.844)	0.7082
PSA density	0.9925 (0.9558-1.031)	0.6935
Clinical T stage	1.235 (0.2377-6.421)	0.8015
Maximum tumor diameter	1.080 (1.024-1.138)	0.004351*
nADCmean	1.074 (0.9714-1.188)	0.1629

Patients with higher clinical T stage (T4 vs T3) did not show a trend toward worse survival. On the other hand, MTD appeared to stratify survival clearly. The group with larger tumors (≥49 mm) had a shorter median overall survival (51 months, 95% CI, 18 not available (NA)) compared to those with tumors <49 mm (median overall survival, 95 months, 95% CI, 67-NA), and the difference was statistically significant (p = 0.015). HR was 1.080 (95% CI, 1.024-1.138). Higher nADCmean (≥0.19) was a prognostic factor of disease progression (p = 0.031). Median progression-free survival was 22.5 months (95% CI, 10-72) and NA (95% CI, 51-NA). HR was 1.068 (95% CI, 0.994-1.147).

## Discussion

In this retrospective analysis of 19 patients with metastatic PCa, we examined whether MRI-derived tumor characteristics prior to treatment could be a prognostic factor for patient survival and disease progression. We found that MTD was associated with poorer overall survival, while clinical T stage did not show a trend toward worse survival. The higher nADCmean was a prognostic factor of disease progression. GS and PSA density were not strong discriminators of outcome.

Prostate MRI has been widely used to diagnose and stage PCa in clinical practice [4-6,12,13,21,25], and clinical T stage is associated with biochemical recurrence, systemic progression, and cancer death after radical prostatectomy [10,11]. However, clinical T stage did not clearly distinguish outcomes in this study. The reason could be that a high clinical T stage was common in metastatic PCa as shown in this study and could not stratify the cohort. On the other hand, MTD stratified survival clearly. MTD is associated with extraprostatic extension, margin positivity, seminal vesicle invasion, lymph node metastasis, and biochemical recurrence after radical prostatectomy [19,20]. Large tumor size or volume is a prognostic factor of biochemical recurrence in PCa patients who underwent radiation therapy [26]. This study showed that MTD was a prognostic factor even in the setting of metastatic PCa and could be more useful for stratification than the clinical T stage.

PSA is widely used for PCa screening. However, the blood PSA level can be affected by the size of the prostate gland. PSA density is typically calculated by determining the ratio between the blood PSA level and the estimated prostate volume before treatment [27]. PSA density was initially used to differentiate between benign prostatic hypertrophy and PCa [28]. Furthermore, PSA density is a useful predictor for local invasion, lymph node and bone metastasis, and biochemical recurrence of PCa [22,27,29-32]. However, it was not a strong discriminator of outcome in the setting of metastatic PCa in this study. PSA density might be less useful as a prognostic factor of metastatic PCa. This finding needs to be validated in a larger study.

ADC negatively correlates with GS and cell density [14-18] and is associated with D'Amico clinical risk scores and the Ki-67 positivity rate of PCa [17,18]. Contrary to the expectation that lower nADCmean would indicate worse prognosis, higher nADCmean was associated with disease progression in this study. As GS was mainly 8 or 9, PCa with a characteristic that raises ADC might have led to this result. Considering negative correlation between ADC and cell density [14], relatively low cell density for high GS could be one of the factors. ADC could also increase by heterogeneous tumor morphology [33]. The relationship between cribriform pattern and ADC is complex, and cribriform patterns can demonstrate a positive relationship to ADC [34]. Invasive cribriform growth pattern or intraductal carcinoma in PCa is reported to be associated with poor prognosis [35-37]. Such components might have affected the results. Further studies with a larger number of patients are necessary to validate this finding.

This study has several limitations. First, this was a single-institution retrospective study that included a small number of patients. With only 19 patients, the statistical power to detect differences is low, and results must be interpreted with caution. Second, the diagnosis of BMs was made without pathological evidence; therefore, the possibility of BMs from other malignancies was not ruled out. However, this possibility was mitigated by excluding patients with concomitant or past malignancies. Third, we did not include contrast-enhanced dynamic T1-weighted imaging (T1WI), because not all the patients had undergone dynamic contrast-enhanced T1WI, and the size of study population was limited. Further investigations with more patients and dynamic contrast-enhanced T1WI are required in future studies. Finally, ADC could have been affected by different MRI scanners and imaging parameters. However, we performed normalization for ADC to mitigate the effects.

## Conclusions

In conclusion, the present study indicated that even in the setting of metastatic prostate cancer, larger maximum tumor diameter indicates poorer prognosis and could be more useful than clinical T stage as a prognostic factor. Higher normalized mean apparent diffusion coefficient was associated with disease progression. Histopathological characteristics that raise apparent diffusion coefficient despite high Gleason score such as relatively low cell density, heterogeneous morphology, and cribriform pattern might have led to this result. In addition to local assessment, magnetic resonance imaging-derived parameters could help guide treatment planning and risk assessment in metastatic prostate cancer. However, this study included a small number of patients, and the results should be interpreted cautiously. Further studies with a larger number of patients are necessary to validate these findings.
